# Do Health Care Providers Use Online Patient Ratings to Improve the Quality of Care? Results From an Online-Based Cross-Sectional Study

**DOI:** 10.2196/jmir.5889

**Published:** 2016-09-19

**Authors:** Martin Emmert, Nina Meszmer, Uwe Sander

**Affiliations:** ^1^ Health Services Management, Institute of Management School of Business and Economics Friedrich-Alexander-University Erlangen-Nuremberg Nuremberg Germany; ^2^ Health Care Management, Institute of Management School of Business and Economics Friedrich-Alexander-University Erlangen-Nuremberg Nuremberg Germany; ^3^ Media, Information and Design Department of Information and Communication University of Applied Sciences and Arts, Hannover Hannover Germany

**Keywords:** public reporting, physician-rating website, quality measures, patient care, quality of health care

## Abstract

**Background:**

Physician-rating websites have become a popular tool to create more transparency about the quality of health care providers. So far, it remains unknown whether online-based rating websites have the potential to contribute to a better standard of care.

**Objective:**

Our goal was to examine which health care providers use online rating websites and for what purposes, and whether health care providers use online patient ratings to improve patient care.

**Methods:**

We conducted an online-based cross-sectional study by surveying 2360 physicians and other health care providers (September 2015). In addition to descriptive statistics, we performed multilevel logistic regression models to ascertain the effects of providers’ demographics as well as report card-related variables on the likelihood that providers implement measures to improve patient care.

**Results:**

Overall, more than half of the responding providers surveyed (54.66%, 1290/2360) used online ratings to derive measures to improve patient care (implemented measures: mean 3.06, SD 2.29). Ophthalmologists (68%, 40/59) and gynecologists (65.4%, 123/188) were most likely to implement any measures. The most widely implemented quality measures were related to communication with patients (28.77%, 679/2360), the appointment scheduling process (23.60%, 557/2360), and office workflow (21.23%, 501/2360). Scaled-survey results had a greater impact on deriving measures than narrative comments. Multilevel logistic regression models revealed medical specialty, the frequency of report card use, and the appraisal of the trustworthiness of scaled-survey ratings to be significantly associated predictors for implementing measures to improve patient care because of online ratings.

**Conclusions:**

Our results suggest that online ratings displayed on physician-rating websites have an impact on patient care. Despite the limitations of our study and unintended consequences of physician-rating websites, they still may have the potential to improve patient care.

## Introduction

Over the last years, physician-rating websites (PRWs) have become a popular tool to create more transparency surrounding the quality of health care providers in the United States and other industrialized countries [1-3]. They usually provide structural information about a physician’s office as well as online-derived patient satisfaction results [3]. Regarding the latter, the rating systems usually contain both scaled-rating systems (eg, stars, grades) and narrative comments. Although scaled-rating systems with standardized questions present a more structured way to receive answers about different aspects of care [4], free-text commentaries allow patients to express themselves in their own words [5]. The comments are intended to provide a more complete picture of the patient experience with that provider, incorporating emotional reactions and the meaning that patients ascribe to their experiences [4,6]. When comparing those two features, narrative comments are meant to be more powerful [7] because users are drawing more attention to words than to numbers [8]. Furthermore, narrative comments have been suggested as one possibility to provide performance metrics that are more easily understood, raising the willingness of users to provide a substantial feedback and creating a more personal feedback than other rating formats [4,9,10].

In general, PRWs and other established public reporting instruments (eg, the Wisconsin Collaborative for Healthcare Quality [11], the New York State Coronary Artery Bypass Surgery Report [12], or Nursing Home Compare [13]) intend to improve the quality of health care via two different pathways. According to the first pathway (“selection”), health care services are shifted to caregivers who provide better quality of care. However, there seems to be little or no impact on the selection of health care providers by patients and families or their representatives so far [14]. Nevertheless, a previously published cross-sectional study has shown that 65% of PRW users consulted a particular physician based on the online ratings and 52% avoided consulting a particular physician because of the ratings [15]. In addition, the second pathway (“changes”) describes a mechanism by which providers are motivated to improve their quality of care for patients [16]. Regarding results from the latest systematic reviews, it seems that publicly releasing performance data stimulates quality improvement activity by means of offering new services, changing policies, change in personnel, and an increase in quality improvement activities [14,17].

So far, most of the evidence on whether public reporting instruments improve the quality of care comes from the inpatient sector [12,18,19]; little evidence is available in the outpatient sector [8,11,20]. Even further, no evidence is available on whether PRWs have an impact on the quality of care by motivating physicians to improve their quality of care [21]. In addition, little is known about the characteristics of physicians who use online rating websites or for what purposes [22]. Therefore, it is important to gain a scientific understanding of whether online-based rating websites have the potential to contribute to a better standard of care. In this paper, we present the results of an online-based cross-sectional study that examines (1) which health care providers use online rating websites (2) for what purposes and (3) assesses whether health care providers use online patient ratings to improve patient care.

## Methods

### Design and Data Source

We conducted an online-based cross-sectional study by surveying outpatient physicians and other outpatient health care providers (eg, midwives, speech and language therapists) who have registered on the German PRW jameda (September 2015). These providers either subscribed to a monthly newsletter that contained an overview of all individual ratings posted on jameda over the previous four weeks or booked a jameda service product. On jameda, providers can register for a free-of-charge basic account that permits them to modify their personal or structural office data and ensures that they receive notification of online ratings on a monthly basis, as well as including the possibility of commenting on the patients’ ratings on the website. Three products are offered on jameda that contain different tools, such as special presentation formats of the practice or the uploading of additional data. To date, jameda is likely to play the most important role in the German PRW movement [23]. For our purposes, the Web service provider of jameda sent out a special issue newsletter via email to registered health care providers (N=25,000) that contained an invitation and an online link to participate in the survey. The newsletter also contained some information about the study and its purpose. As an incentive, we held drawings for four Amazon vouchers with a value of €150 each.

We designed the survey by using QuestBack’s Internet-based Enterprise Feedback Suite survey software. The questionnaire contained 26 questions about PRWs in general and consisted of three parts. After collecting sociodemographic information, the second part asked questions about the knowledge and usage of PRWs. In the third part of the survey, we aimed to assess whether and how health care providers react to online ratings and whether they had implemented any of 20 listed measures in order to improve patient care. Those measures were derived from a systematic review regarding the impact of public reporting on the quality of patient care (described subsequently). We distinguished between scaled-survey questions and narrative comments to investigate which are of greater importance to the providers (see Multimedia Appendix 1 for the developed survey instrument.)

Before conducting the study, the questionnaire was piloted by 25 individuals to ensure the proper working of the survey, the randomization process of some questions, and the comprehensibility of the wording. The pretest resulted in minor adaptations to the wording of some questions. We conducted a systematic search procedure in the databases Medline (via PubMed) and the Cochrane Library (May 2015) to identify studies that have researched the impact of public reporting on the provider behavior with respect to the implementation of quality measures. Our search yielded 12 studies that were related to the outpatient (n=3) or inpatient sector (n=9). However, no study had investigated the usage and impact of online rating websites on provider behavior.

### Data Analysis

Results are presented as both mean and standard deviation for continuous variables and as numbers and percentages for categorical variables. We performed comparisons between more than two groups by using a chi-square test (two-sided) for categorical variables and applying the Kruskal-Wallis test for continuous nonparametric variables. The Shapiro-Wilk test was applied to examine the normality of the data distribution. Multilevel multivariate logistic regression models were used to assess how much of the providers’ reaction to implement measures could be explained by demographic and online report card-related variables. The dependent variable indicated whether a health care provider had implemented any measure(s) to improve patient care (yes/no). Regarding the independent variables, we used the following sequence of models: (1) adjusted for demographics (age, gender, marital status, Internet use, medical specialty), (2) adding information regarding any booked jameda service products (yes/no), and (3) adding online report card-related issues (ie, frequency of use, appraisal of the trustworthiness of the online ratings). All statistical analyses were conducted using SPSS version 22.0 (IBM Corp, Armonk, NY, USA). Observed differences were considered statistically significant if *P*<.05.

## Results

In total, our final study sample consisted of 2360 respondents who completed the survey (response rate=9.44%, 2360/25000; completion rate=67.29%, 2360/3507). We excluded 49 participants from subsequent analysis because of extremely short answer times and/or inconsistent answer patterns. The completed online surveys took a mean 9.63 (SD 9.03) minutes. The overall mean age was 49.63 (SD 8.69) years, 66.67% (1560/2340) respondents were male, and the mean duration of practice in the doctors’ office was 12.99 (SD 9.10) years (Table 1).

Regarding the medical specialty, 17.50% (413/2360) of the surveyed sample were general practitioners, 69.36% (1637/2360) were specialists, and 13.14% (310/2360) were other health care-related professions (eg, midwives). The three groups did not differ significantly in terms of age and Internet use, but did in terms of gender, marital status, and years of practice in the office.

### Awareness and Usage of Online Rating Websites

Most providers became aware of the websites through the Internet (71.91%, 1697/2360), contact with the providers of PRWs (20.08%, 474/2360), or advertisements (16.74%, 395/2360) (Table 2). Differences regarding the sources of awareness between the three provider groups (ie, general practitioners, specialists, others) could all be shown to be statistically significant (*P*<.05 each), except for recommendations by friends and relatives as well as others. Furthermore, specialists used PRWs more frequently than general practitioners and other providers did (*P*<.001). Most providers (87.08%, 2055/2360) used online rating websites to read comments for their individual practice. Almost half of all providers read comments for other providers (48.69%, 1149/2360) and slightly more than one in three providers (35.97%, 849/2360) to know which measures might be implemented to increase patient satisfaction. In addition, 12.08% (285/2360) of respondents stated that they used online ratings for referring patients to other providers. Therefore, numbers for general practitioners (12.1%, 50/413) and specialists (10.38%, 170/1637) were significantly lower than those for other providers (21.0%, 65/310, *P*<.001). In addition, specialists evaluated their ratings significantly more often than general practitioners and other providers did (*P*<.001). Most providers evaluated the ratings themselves (84.87%, 2003/2360).

**Table 1:** Characteristics of respondents according to their medical discipline.

Characteristics			Overall (N=2360)	General practitioners (n=413)	Specialists (n=1637)	Others (n=310)	*P*^a^ value
**Age (years)**							
	Mean (SD)		49.63 (8.69)	50.61 (8.65)	49.41 (8.76)	49.45 (8.33)	.08
	**Ranges, n (%)**						.06
		<35	109 (5.05)	9 (2.4)	87 (5.83)	13 (4.5)	
		36-45	598 (27.71)	110 (29.0)	411 (27.55)	77 (26.8)	
		46-55	890 (41.24)	142 (37.5)	619 (41.49)	129 (45.0)	
		56-65	494 (22.89)	104 (27.4)	330 (22.12)	60 (20.9)	
		≥66	67 (3.10)	14 (3.7) 45 (3.02)	8 (2.8)		
**Gender, n (%)**							<.001
	Male		1560 (66.67)	278 (67.8)	1175 (72.31)	107 (35.1)	
	Female		780 (33.33)	132 (32.2)	450 (27.69)	198 (64.9)	
**Marital status, n (%)**							<.001
	Married		1706 (77.86)	316 (81.9)	1218 (79.87)	172 (61.4)	
	Widowed		20 (0.91)	10 (2.6)	6 (0.39)	4 (1.4)	
	Single		294 (13.42)	31 (8.0)	206 (13.51)	57 (20.6)	
	Divorced		171 (7.80)	29 (7.5)	95 (6.23)	47 (16.8)	
**Length of practice in the doctor’s office (years)**						
	Mean (SD)		12.99 (9.10)	14.35 (9.67)	12.93 (8.95)	11.48 (8.83)	<.001
	**Ranges, n (%)**						<.001
		<5	616 (27.76)	97 (24.8)	427 (27.80)	92 (31.5)	
		6-10	479 (21.59)	78 (20.0)	323 (21.03)	78 (26.7)	
		11-15	333 (15.01)	56 (14.3)	238 (15.49)	39 (13.4)	
		16-20	262 (11.81)	33 (8.4)	191 (12.43)	38 (13.0)	
		21-25	303 (13.65)	72 (18.4)	211 (13.74)	20 (6.9)	
		≥26	226 (10.18)	55 (14.1)	146 (9.51)	25 (8.6)	
**Internet use, n (%)**							.41
	Several times a day		2124 (90.00)	366 (88.6)	1485 (90.71)	273 (88.1)	
	Once a day		149 (6.31)	27 (6.5)	98 (5.99)	24 (7.7)	
	Less than once a day		87 (3.69)	20 (4.8)	54 (3.30) 13 (4.2)		
**Jameda service product, n (%)**							<.001
	Basic product		1601 (67.84)	365 (88.4)	1031 (62.98)	205 (66.1)	
	Any service product (eg, gold, silver, platinum)		759 (32.16)	48 (11.6)	606 (37.02)	105 (33.9)	

^a^*P* value was calculated using Kruskal-Wallis [1] and chi-square test [2].

**Table 2:** Awareness and use of physician-rating websites.

Characteristics		Overall, n (%) (N=2360)	General practitioners, n (%) (n=413)	Specialists, n (%) (n=1637)	Others, n (%) (n=310)	*P*^a^ value
**How did you become aware of physician-rating websites?**						
	Contact with physician-rating website provider	474 (20.08)	78 (18.9)	352 (21.50)	44 (14.2)	.01
	Internet	1697 (71.91)	295 (71.4)	1198 (73.18)	204 (65.8)	.03
	Contact with patients	292 (12.37)	58 (14.0)	210 (12.83)	24 (7.7)	.02
	Recommendations by peers	281 (11.91)	15 (3.6)	210 (12.83)	56 (18.1)	<.001
	Advertisement	395 (16.74)	66 (16.0)	300 (18.33)	29 (9.4)	<.001
	Newspapers or magazines	141 (5.97)	29 (7.0)	112 (6.84)	0 (0)	<.001
	Recommendations by friends or relatives	89 (3.77)	12 (2.9)	61 (3.73)	16 (5.2)	.29
	Others	111 (4.70)	23 (5.6)	79 (4.83)	9 (2.9)	.23
**How often do you use physician-rating websites?**						<.001
	At least once per day	155 (6.57)	16 (3.9)	130 (7.94)	9 (2.9)	
	Several times a week	273 (11.57)	29 (7.0)	209 (12.77)	35 (11.3)	
	Once per week	500 (21.19)	92 (22.3)	346 (21.14)	62 (20.0)	
	Once per month	655 (27.75)	121 (29.3)	445 (27.18)	89 (28.7)	
	Less frequently	640 (27.12)	129 (31.2)	417 (25.47)	94 (30.3)	
	Never	137 (5.81)	26 (6.3)	90 (5.50)	21 (6.8)	
**For what purpose(s) do you use physician-rating websites?**						
	Reading own ratings	2055 (87.08)	363 (87.9)	1448 (88.45)	244 (78.7)	<.001
	Commenting on own ratings	656 (27.80)	94 (22.8)	506 (30.91)	56 (18.1)	<.001
	Reading ratings of other physicians because of interest	1149 (48.69)	181 (43.8)	816 (49.85)	152 (49.013)	.09
	Readings ratings of other physicians for patient referral	285 (12.08)	50 (12.1)	170 (10.38)	65 (21.0)	<.001
	Own practice marketing	785 (33.26)	78 (18.9)	579 (35.37)	128 (41.3)	<.001
	I use physician-rating websites for other purposes	68 (2.88)	12 (2.9)	46 (2.81)	10 (3.23)	.92
**How often do you evaluate your ratings on physician-rating websites?**						<.001
	At least once per week	443 (18.77)	44 (10.7)	348 (21.26)	51 (16.5)	
	Several times a month	236 (10.00)	29 (7.0)	174 (10.63)	33 (10.7)	
	Once per month	765 (32.42)	142 (34.4)	535 (32.68)	88 (28.4)	
	Less frequently	734 (31.10)	161 (39.0)	477 (29.14)	96 (31.0)	
	Never	182 (7.71)	37 (9.0)	103 (6.29)	42 (13.6)	
**Who is responsible for evaluating the online ratings for your practice?**						
	I evaluate the online ratings for my practice myself	2003 (84.87)	354 (85.7)	1385 (84.61)	264 (85.2)	.84
	Medical assistant(s)	115 (4.87)	24 (5.8)	89 (5.44)	2 (0.7)	<.001
	Practice manager	180 (7.63)	17 (4.1)	159 (9.71)	4 (1.3)	<.001
	Others	47 (1.99)	6 (1.5)	41 (2.50)	0 (0)	.01

^a^*P* value was calculated using chi-square test for all variables.

### Implemented Measures to Improve Patient Care Because of Online Ratings

Overall, 54.66% (1290/2360) of respondents stated that they had used online ratings to implement some measure(s) to improve patient care (Tables 3 and 4). Numbers for specialists (57.79%, 946/1637) were significantly higher than those for general practitioners (50.1%, 207/413) and other providers (44.2%, 137/310, *P*<.001). The most widely implemented measures were related to communication with patients (28.77%, 679/2360) or the appointment scheduling process (23.60%, 557/2360). Staff-related measures also played an important role; 10.38% (245/2360) of respondents stated that they had invested in further training of staff and 5.89% (139/2360) had recruited additional staff. Scaled-survey results seemed to have a greater impact on developing measures to improve patient care. The results showed that half of respondents (50.08%, 1182/2360) developed measures from scaled-survey ratings, whereas only 43.90% (1036/2360) stated that they had used information from narrative comments. In line with these results, specialists made significantly more use of the information in both scaled-survey ratings and narrative comments (53.21%, 871/1637 and 47.16%, 772/1637, respectively) than general practitioners (44.6%, 184/413 and 41.7%, 172/413, respectively) and other providers (41.0%, 127/310 and 29.7%, 92/310, respectively; *P*<.001).

**Table 3:** Measures that were implemented to increase patient satisfaction because of online ratings by results type and discipline (N=2360).

Measures	Overall by results type, n (%)			Overall by discipline, n (%)			
	Overall	Scaled-survey results	Narrative comments results	General practitioners (n=413)	Specialists (n=1637)	Others (n=310)	*P*^a^ value
Improvement of the communication with patients	679 (28.77)	527 (22.33)	444 (18.81)	107 (25.9)	517 (31.58)	55 (17.7)	<.001
Improve appointment scheduling process	557 (23.60)	456 (19.32)	317 (13.43)	89 (21.6)	423 (25.84)	45 (14.5)	<.001
Change in office workflow	501 (21.23)	379 (16.06)	310 (13.14)	88 (21.3)	378 (23.09)	35 (11.3)	<.001
Improvement of the waiting room equipment	266 (11.27)	225 (9.53)	135 (5.72)	42 (10.2)	202 (12.34)	22 (7.1)	.02
Training of the staff	245 (10.38)	173 (7.33)	152 (6.44)	39 (9.4)	196 (11.97)	10 (3.2)	<.001
Reassigning staff responsibilities	231 (9.79)	152 (6.44)	144 (6.10)	38 (9.2)	185 (11.30)	8 (2.6)	<.001
Investments in new technologies/ equipment (IT^b^ equipment)	200 (8.47)	162 (6.86)	104 (4.41)	31 (7.5)	150 (9.16)	19 (6.1)	.16
Expand office hours	189 (8.01)	155 (6.57´)	104 (4.41)	26 (6.3)	132 (8.06)	31 (10.0)	.19
Introduction of patient reminders (eg, email reminders)	184 (7.80)	145 (6.14)	91 (3.86)	23 (5.6)	141 (8.61)	20 (6.5)	.08
Further educational training myself	157 (6.65)	113 (4.79)	90 (3.81)	14 (3.4)	102 (6.23)	41 (13.2)	<.001
Recruitment of additional staff	139 (5.89)	115 (4.87)	75 (3.18)	22 (5.3)	109 (6.66)	8 (2.6)	.02
Improvement of the communication with other providers	108 (4.58)	77 (3.26)	55 (2.33)	14 (3.4)	81 (4.95)	13 (4.2)	.38
Introduction of guidelines	100 (4.24)	76 (3.22)	51 (2.16)	2 (0.5)	83 (5.07)	15 (4.8)	<.001
Dismissing staff	78 (3.31)	56 (2.37)	46 (1.95)	12 (2.9)	61 (3.73)	5 (1.6)	.14
Higher usage of guidelines	77 (3.26)	57 (2.42)	39 (1.65)	10 (2.4)	58 (3.54)	9 (2.9)	.48
Planning of follow-up tests	65 (2.75)	46 (1.95)	35 (1.48)	15 (3.6)	44 (2.69)	6 (1.9)	.37
Hygiene improvement measures	64 (2.71)	48 (2.03)	33 (1.40)	9 (2.2)	46 (2.81)	9 (2.9)	.76
Others	140 (5.93)	86 (3.64)	108 (4.58)	22 (5.3)	97 (5.93)	21 (6.8)	.72
I have not implemented any measures	1070 (45.34)	1178 (49.92)	1324 (56.10)	206 (49.9)	691 (42.21)	173 (55.8)	<.001

^a^*P* value was calculated using chi-square test.

^b^IT: Information Technology.

**Table 4:** Measures that were implemented to increase patient satisfaction because of online ratings by scaled-survey and negative comments results (N=2360).

Measures	Scaled-survey results, n (%)				Narrative comments results, n (%)			
	General practitioners (n=413)	Specialists (n=1637)	Others (n=310)	*P* ^a^	General practitioners (n=413)	Specialists (n=1637)	Others (n=310)	*P* value
Improvement of the communication with patients	83 (20.1)	399 (24.37)	45 (14.5)	<.001	73 (17.7)	341 (20.83)	30 (9.7)	<.001
Improve appointment scheduling process	65 (15.7)	355 (21.69)	36 (11.6)	<.001	54 (13.1)	239 (14.60)	24 (7.7)	.005
Change in office workflow	68 (16.5)	281 (17.17)	30 (9.7)	.004	55 (13.3)	238 (14.54)	17 (5.5)	<.001
Improvement of the waiting room equipment	37 (9.0)	169 (10.32)	19 (6.1)	.06	23 (5.6)	103 (6.29)	9 (2.9)	.06
Training of the staff	32 (7.8)	136 (8.31)	5 (1.6)	<.001	25 (6.1)	122 (7.45)	5 (1.6)	<.001
Reassigning staff responsibilities	24 (5.8)	122 (7.45)	6 (1.9)	<.001	26 (6.3)	115 (7.03)	3 (1.0)	<.001
Investments in new technologies/ equipment (IT^b^ equipment)	22 (5.3)	124 (7.57)	16 (5.2)	.12	19 (4.6)	80 (4.89)	5 (1.6)	.04
Expand office hours	22 (5.3)	109 (6.66)	24 (7.7)	.42	12 (2.9)	74 (4.52)	18 (5.8)	.16
Introduction of patient reminders (eg, email reminders)	21 (5.1)	109 (6.66)	15 (4.8)	.29	14 (3.4)	69 (4.22)	8 (2.6)	.34
Further educational training myself	9 (2.2)	71 (4.34)	33 (10.7)	<.001	9 (2.2)	63 (3.85)	18 (5.8)	.04
Recruitment of additional staff	18 (4.4)	91 (5.56)	6 (1.9)	.02	10 (2.4)	60 (3.67)	5 (1.6)	.11
Improvement of the communication with other providers	9 (2.2)	58 (3.54)	10 (3.2)	.38	9 (2.2)	42 (2.57)	4 (1.3)	.38
Introduction of guidelines	2 (0.5)	63 (3.85)	11 (3.6)	.002	1 (0.2)	41 (2.50)	9 (2.9)	.01
Dismissing staff	10 (2.4)	43 (2.63)	3 (1.0)	.21	6 (1.5)	37 (2.26)	3 (1.0)	.23
Higher usage of guidelines	7 (1.7)	42 (2.57)	8 (2.6)	.58	6 (1.5)	31 (1.89)	2 (0.7)	.27
Planning of follow-up tests	8 (1.9)	32 (1.95)	6 (1.9)	.99	9 (2.2)	24 (1.47)	2 (0.7)	.24
Hygiene improvement measures	8 (1.9)	33 (2.02)	7 (2.3)	.96	5 (1.2)	25 (1.53)	3 (1.0)	.70
Others	13 (3.2)	57 (3.48)	16 (5.2)	.30	17 (4.1)	76 (4.64)	15 (4.8)	.88
I have not implemented any measures	229 (55.5)	766 (46.79)	183 (59.0)	<.001	241 (58.4)	865 (52.84)	218 (70.3)	<.001

^a^*P* value was calculated using chi-square test.

^b^IT: Information Technology.

Table 5 presents the results for implementing measures to improve patient care according to the medical specialty (here shown for medical disciplines with at least 20 providers and the seven most frequently implemented measures). As displayed, ophthalmologists (68%, 40/59) and gynecologists (65.4%, 123/188) were most likely to implement any measure to improve patient care. In contrast, the lowest percentages were calculated for psychiatrists (38%, 29/77) and pediatrics (40%, 16/40). Thereby, the mean number of implemented measures (mean 3.06, SD 2.29) was calculated to range between 1.81 (SD 1.05) for pediatrics and 4.29 (SD 3.05) for urologists, respectively. The association between the two variables could be shown to be marginally significant (Spearman *P*=.47 and *P*=.07, respectively).

**Table 5:** An overview of the seven most relevant measures that were implemented to improve patient care because of online ratings according to the medical specialty (medical disciplines with n>20; N=2360) (see Multimedia Appendix 2 for a complete overview).

Medical discipline	Any measure implemented, n (%)	Mean measures, mean (SD)	Measure, n (%)^a^						
			M1	M2	M3	M4	M5	M6	M7
Ophthalmology	40 (67.80)	3.48 (2.61)	20 (33.90)	23 (38.98)	17 (28.81)	13 (22.03)	11 (18.64)	13 (22.03)	6 (10.17)
Gynecology/obstetrics	123 (65.43)	3.29 (2.28)	58 (30.85)	59 (31.38)	57 (30.32)	26 (13.83)	29 (15.43)	26 (13.83)	18 (9.57)
Physical and rehabilitative medicine	15 (65.22)	2.43 (1.83)	7 (30.43)	3 (13.04)	6 (26.09)	2 (8.70)	1 (4.35)	2 (8.70)	1 (4.35)
Otorhinolaryngology (ENT^b^)	59 (62.11)	3.44 (2.42)	38 (40.00)	25 (26.32)	28 (29.47)	10 (10.53)	11 (11.58)	13 (13.68)	16 (16.84)
Neurosurgery	17 (60.71)	3.07 (1.62)	12 (42.86)	10 (35.71)	6 (21.43)	1 (3.57)	4 (14.29)	2 (7.14)	2 (7.14)
Surgery/orthopedists	140 (60.61)	3.02 (2.27)	75 (32.47)	70 (30.30)	70 (30.30)	30 (12.99)	29 (12.55)	29 (12.55)	20 (8.66)
Oral and maxillofacial surgery	21 (60.00)	3.45 (1.21)	10 (28.57)	13 (37.14)	9 (25.71)	6 (17.14)	3 (8.57)	2 (5.71)	6 (17.14)
Urology	38 (58.46)	4.29 (3.05)	25 (38.46)	23 (35.38)	22 (33.85)	8 (12.31)	10 (15.38)	15 (23.08)	6 (9.23)
Dentistry	350 (57.47)	3.12 (2.45)	197 (32.35)	127 (20.85)	113 (18.56)	84 (13.79)	77 (12.64)	61 (10.02)	60 (9.85)
Dermatology and sexually transmitted diseases	41 (55.41)	2.79 (1.91)	24 (32.43)	21 (28.38)	14 (18.92)	4 (5.41)	8 (10.81)	8 (10.81)	3 (4.05)
Internal medicine (specialist)	45 (53.57)	2.93 (2.10)	24 (28.57)	26 (30.95)	20 (23.81)	7 (8.33)	6 (7.14)	9 (10.71)	9 (10.71)
General medicine	158 (52.32)	2.94 (2.08)	81 (26.82)	66 (21.85)	67 (22.19)	34 (11.26)	31 (10.26)	31 (10.26)	28 (9.27)
Psychosomatic medicine and psychotherapy	18 (50.00)	3.22 (2.44)	9 (25.00)	7 (19.44)	3 (8.33)	6 (16.67)	2 (5.56)	3 (8.33)	4 (11.11)
Internal medicine (GP^c^)	40 (45.45)	3.10 (2.15)	21 (23.86)	22 (25.00)	22 (25.00)	5 (5.68)	10 (11.36)	8 (9.09)	4 (4.55)
Alternative practitioner	120 (42.25)	2.70 (2.14)	49 (17.25)	38 (13.38)	28 (9.86)	19 (6.69)	10 (3.52)	7 (2.46)	17 (5.99)
Pediatrics and adolescent medicine	16 (40.00)	1.81 (1.05)	10 (25.00)	6 (15.00)	3 (7.50)	4 (10.00)	1 (2.50)	1 (2.50)	0 (0.00)
Psychiatry and psychotherapy	29 (37.66)	2.61 (1.64)	16 (20.78)	13 (16.88)	10 (12.99)	6 (7.79)	5 (6.49)	0 (0.00)	3 (3.90)
Total	1290 (54.66)	3.06 (2.29)	679 (28.77)	557 (23.60)	501 (21.23)	266 (11.27)	245 (10.38)	231 (9.79)	200 (8.47)

^a^ M1=Improvement of the communication with patients; M2=improve appointment scheduling process; M3=change office workflow; M4=improvement of the waiting room equipment; M5=training of the staff; M6=reassigning staff responsibilities; M7=investments in new technologies/equipment.

^b^ENT: ear, nose, and throat.

^c^GP: general practitioner.

**Table 6:** Multivariate regression analyses, including adjusted odds ratio (AOR), 95% confidence interval (CI), and *P* values, of the association between the implementation of measures to increase patient satisfaction because of online ratings (both scaled-survey ratings and narrative comments) and independent variables.

Characteristics		Model 1^a^		Model 2^b^		Model 3^c^	
		AOR (95% CI)	*P* value	AOR (95% CI)	*P* value	AOR (95% CI)	*P* value
**Age (years)**			.57		.71		.85
	<35 (ref)							
	36-45	0.76 (0.49-1.19)	.23	0.77 (0.49-1.20)	.24	0.85 (0.53-1.36)	.50
	46-55	0.77 (0.50-1.19)	.24	0.80 (0.52-1.25)	.33	0.91 (0.57-1.45)	.69
	56-65	0.75 (0.47-1.19)	.22	0.79 (0.50-1.25)	.32	0.90 (0.55-1.47)	.67
	≥66	0.57 (0.29-1.10)	.10	0.63 (0.32-1.23)	.18	0.70 (0.35-1.42)	.32	
**Gender**							
	Male (ref)						
	Female	1.21 (0.99-1.48)	.07	1.16 (0.95-1.43)	.15	1.09 (0.88-1.36)	.43
**Marital status**			.38		.43		.55
	Married (ref)						
	Widowed	1.94 (0.60-5.46)	.21	1.99 (0.71-5.59)	.20	1.32 (0.46-3.80)	.61
	Single	1.14 (0.86-1.50)	.37	1.13 (0.85-1.49)	.40	1.18 (0.88-1.59)	.27
	Divorced	1.20 (0.85-1.69)	.31	1.15 (0.81-1.62)	.44	1.20 (0.83-1.74)	.34
**Internet use**			.70		.77		.21
	Several times a day (ref)						
	Once a day	1.06 (0.72-1.56)	.77	1.13 (0.76-1.66)	.55	1.39 (0.92-2.10)	.12
	Less than once a day	0.82 (0.50-1.35)	.44	0.91 (0.55-1.50)	.71	1.29 (0.75-2.24)	.36
**Medical specialty**			<.001		<.001		<.001
	General practitioner (ref)						
	Specialist	1.31 (1.04-1.66)	.03	1.16 (0.91-1.48)	.23	1.17 (0.91-1.51)	.23
	Others	0.77 (0.55-1.08)	.13	0.68 (0.48-0.96)	.03	0.59 (0.41-0.85)	.004
**Jameda service product**							
	Basic product (ref)						
	Any service product (eg, gold, silver, platinum)			1.61 (1.32-1.96)	<.001	1.13 (0.90-1.41)	.29
**Use of physician-rating websites (frequency)**							<.001
	At least once per day (ref)							
	Several times a week					1.39 (0.86-2.23)	.18
	Once per week					0.98 (0.63-1.51)	.91
	Once per month					0.59 (0.38-0.90)	.01
	Less frequently					0.39 (0.25-0.61)	<.001
	Never					0.18 (0.09-0.34)	<.001
**Appraisal of the trustworthiness of scaled-rating results**							.02
	Not at all trustworthy (ref)							
	Not trustworthy					1.68 (1.01-2.84)	.06
	More or less trustworthy					1.97 (1.17-3.33)	.01
	Somewhat trustworthy					1.93 (1.12-3.31)	.02
	Very trustworthy					1.16 (0.59-2.26)	.67
**Appraisal of the trustworthiness of narrative comments**							.09
	Not at all trustworthy (ref)							
	Not trustworthy					2.11 (1.21-3.69)	.009
	More or less trustworthy					1.89 (1.08-3.28)	.03
	Somewhat trustworthy					1.97 (1.13-3.44)	.02
	Very trustworthy					2.26 (1.22-4.19)	.01

^a^ Model 1: Adjusted for demographics (age, gender, marital status, Internet use, medical specialty) (χ^2^_12_=28,891, *P*=.004, Nagelkerke *R*^2^=.019).

^b^ Model 2: Adjusted for demographics, jameda service product (χ^2^_13_=50,980, *P*<.001, Nagelkerke *R*^2^=.034).

^c^ Model 3: Adjusted for demographics, jameda service product, use of physician-rating websites, appraisal of the trustworthiness of scaled-rating results/narrative comments (χ^2^_26_=251,463, *P*<.001, Nagelkerke *R*^2^=.160).

Multilevel logistic regression models were performed to ascertain the effects of providers’ demographics as well as report card-related variables on the likelihood that providers implemented measures to improve patient care (Table 6). Thereby, the dependent variable indicates whether a health care provider had implemented any measure(s) to improve patient care (yes/no).

All models revealed medical specialty to be a significantly associated predictor (*P*<.001 each). Thereby, the higher odds for specialists were proven to be statistically significant in our baseline model (AOR 1.31, 95% CI 1.04-1.66, *P*=.03). In contrast, the lower odds for other providers were proven to be statistically significant in our more comprehensive models and ranged between AOR 0.59 (95% CI 0.41-0.85, *P*<.001) and AOR 0.68 (95% CI 0.48-0.96, *P*=.03). In addition, the frequency of report card use (*P*<.001) and the appraisal of the trustworthiness of scaled-survey ratings (*P=*.02) were determined to be significantly associated predictors. Further regression analyses were run to determine whether the results differed when related only to implemented measures because of scaled-survey ratings or narrative comments (see Multimedia Appendices 3 and ). As presented, the results could be shown to be very robust in terms of significantly associated predictors.

## Discussion

The aim of this study was to examine which health care providers use online rating websites and for what purposes, as well as to investigate whether health care providers use online ratings to improve their quality of care. Therefore, we conducted an online-based cross-sectional study by surveying 2360 physicians and other health care providers. Our surveyed sample is slightly younger and contains a lower percentage of female respondents than providers who are registered on jameda or those who work in the German outpatient setting, respectively [24]. However, the distribution of our surveyed physicians across Germany as well as the medical specialties is similar to all physicians in the German outpatient setting (see Multimedia Appendix 5). Nevertheless, our sample may not be representative of all providers in the German outpatient setting despite the similarities in its characteristics. First, compared to the German outpatient setting, approximately 8% (25,000/304,818; 147,948 physicians, 69,370 dentists, and 87,500 other providers) of all providers were invited to participate in this survey, of whom slightly more than 9% completed the survey. Second, providers may differ in further characteristics among those who are registered on jameda and those who are not. For example, those less interested in online-related topics (eg, those without a practice website), or PRWs in general, are less likely to be represented by our results.

Our results show that more than half of the surveyed providers (55%) use online ratings to develop measures to increase the quality of patient care. This result is lower than the findings by Friedberg et al [25], who reported that 83% of interviewed physician group leaders used patient experience to improve the performance in the US outpatient setting. Furthermore, Smith and colleagues [20] showed an increase in the percentage of providers that implemented at least one of 22 possible diabetes improvement interventions from 75% to 94% after the onset of public reporting. The study approach here was quite similar because in both studies a literature-based list of possible interventions was presented. Thereby, the mean number of implemented measures to increase patient care in our study (mean 3.1, SD 2.3) was lower than in the study by Smith et al (mean 8.7, SD 4.5) [20].

The most widely implemented quality measures in our study are related to the communication with patients (28.77%, 679/2360), the appointment scheduling process (23.60%, 557/2360), and the office workflow (21.23%, 501/2360). These findings are in line with the results of Friedberg et al [25], who identified the change in the office workflow (eg, procedures for handling test results or incoming mail) as the most widely implemented initiative (70%), much more common even than in our study. The improvement of the appointment scheduling process was similarly reported by 27% of providers. Other common implemented quality measures in the Friedberg et al study (eg, training nonclinicians: 57%; reassigning staff responsibilities: 45%; or hiring or firing clinicians or staff: 36%) were higher in the US studies. Nevertheless, staff-related measures were implemented by approximately 16% of all respondents in our survey and thus do also play a significant role in the German context. Although 10.38% (245/2360) of the respondents have invested in further training of staff, 5.89% (139/2360) have recruited additional staff, and 3.31% (78/2360) have dismissed staff as a consequence of the online ratings.

Another study showed the adoption of guidelines (87%) and the introduction of patient reminders (82%) to be the most frequently introduced quality measures [11]. Results from our study are far below those percentages and were shown to range between 4% (introduction of guidelines) and 8% (introduction of patient reminders). It is likely that differences in the surveyed samples (ie, individual physicians vs physician group leaders) may account for some of the difference. For example, physician groups in the study of Friedberg et al [25] represent all physicians who work at one or more practices with at least one group-level manager. This manager coordinates the correspondence with health plans and controls the performance of physician groups [25]. Furthermore, successful physician groups share a unified physician-led network, an infrastructure to support group performance, and incentives to engage individual physicians toward group goals [26]. Consequently, these physician groups might be more active in implementing quality measures due to an established infrastructure and developed competencies. In contrast, our sample comprises individual physicians who may not have the infrastructure or the competencies to introduce quality measures on such a large scale. Another study does not present detailed information about the frequency of implemented measures, but states several measures that have been widely implemented, such as the use of phone and mail patient reminders about existing upcoming appointments or to schedule follow-up tests, guideline-based reminders for providers at appointments about services that their patients should receive, or the adoption of care guidelines [20].

As shown, most providers use the websites for reading comments for their individual practice (87.08%, 2055/2360). Of those, 56.69% (1165/2055) stated that they had implemented quality measures because of the online ratings. This also means that 43.31% (890/2055) of those providers did not implement any quality measure(s), possibly because of a lack of time or trust in the online ratings. Others might find it challenging to develop quality measures at all because anonymous ratings sometimes do not provide enough information to learn about quality deficits [27]. Reading other physicians’ ratings out of interest (49%) provides an opportunity for physicians to draw comparisons. This may also have a positive impact on the overall quality of care because some physicians are becoming engaged in implementing quality measures in order to perform better than their colleagues. According to our numbers, 12.08% (285/2360) of the respondents use online ratings for referring patients to other providers. This result exceeds those of other studies, which determined that physicians do not use publicly available quality information [28] or change their referral practices due to public reports [29,30]. The remaining providers might not use online ratings for referral decisions because subjective patient ratings do not accurately display the quality of health care providers [31,32].

We were able to show further that scaled-rating systems seem to have a greater impact on implementing quality measures than narrative comments in absolute terms. This might be because scaled ratings systems offer a more structural approach to rate health care providers in comparison to narrative comments [4]. Therefore, physician-rating systems provide standardized categories that more promptly enable the indication of quality deficits. However, even though narrative comments provide a more complete picture of the patients’ experiences with the health care provider [6], they are more complicated and time-consuming to analyze. For example, it might be easier to determine deficits such as long waiting times for an appointment out of scaled-rating systems. This is because most scaled-rating systems contain this issue, but only 13% of narrative comments address this topic [33].

Finally, even though our results could demonstrate positive effects of public reporting on patient care, several unintended effects should be regarded for the special case of PRWs. For example, public reporting might reduce the access to care because health care providers tend to avoid high-risk patients and prefer low-risk patients (“cherry picking” or “cream skimming”) [29,34]. This effect is likely to be even greater in the case of online rating websites compared to traditional reporting instruments (see previous) because no risk-adjustment of online ratings is carried out [3]. Furthermore, Lindenauer et al [35] mentioned the neglect of more important aspects of care. So far, all PRWs in Germany, and most in the United States, solely contain patient satisfaction as a performance measure. Results for guideline-based treatment (eg, for chronic conditions such as type 2 diabetes ) are not presented on many report cards, meaning ratings for physicians are only based on patient satisfaction scores. From the perspective of a physician, this might be challenging because most (narrative) ratings concern the friendliness and general impression of a physician rather than the medical outcomes [33]. For instance, a physician might neglect the patients’ desire for prescribed antibiotics in the case of a nonbacterial infection. Even though this behavior is correct from a medical point of view, patients might experience this differently and leave a low rating for a physician. Lindenauer et al [35] also stated the unintended risk of an increase in performance rates through coding and documentation (“gaming”). So far, this risk might be of lower importance on most reporting websites because no secondary data are used (which might be gamed). However, online rating websites are vulnerable to other gaming aspects [3,27]. For example, physicians may rate themselves, leave a low rating for their colleagues, or ask the more satisfied patients to leave a rating [27].

Our results demonstrate that health care providers in Germany use PRWs for reading comments for their individual practice. More than half of those providers stated that they had implemented quality measures in order to improve patient care. The most widely implemented quality measures are related to communication with patients, the appointment scheduling process, and office workflow. In addition, staff-related measures also play a significant role in the German context. Scaled-survey results seem to have a greater impact on deriving measures than narrative comments. This might be because the latter are more complicated and time-consuming to analyze. Thus, our results confirm findings from the United States showing that online rating websites may have a positive impact on patient care.

There are some limitations that have to be considered when interpreting the results of this study. First, this study applied a cross-sectional design. Thus, we were able to identify an association between exposure and outcomes, but could not infer cause and effect. Next, even though our sample is representative of those physicians who are registered on the largest German PRW, representation of all practicing health care providers in the outpatient setting in Germany is not achievable (eg, those less interested in online-related topics, those who do not want to deal with this topic in general). In addition, some participants in our sample might be more familiar with Internet-related topics, which may account for some of the high awareness and usage levels of rating websites. Furthermore, the participants were recruited online by the provider of jameda. This means that we only surveyed providers who are active on jameda. Thus, our findings cannot be generalized for providers on other rating websites. For our regression analysis, we used whether providers have implemented any quality measure or not as the binary dependent variable. As shown previously, there were severe differences in the extent of the implementation between the individual measures. Therefore, results might be different if we used the implementation of the individual measures as the dependent variable.

## Appendix

Multimedia Appendix 1 Survey instrument.

Multimedia Appendix 2 Overview of all measures that were implemented to improve patient care because of online ratings according to the medical specialty (medical disciplines with N>20) (N=2360).

Multimedia Appendix 3 Multivariate regression analyses; adjusted odds ratio (OR), 95% confidence interval (CI), and *P* value of the association between the implementation of measures to increase patient satisfaction because of scaled survey online ratings and independent variables.

Multimedia Appendix 4 Multivariate regression analyses; adjusted odds ratio (OR), 95% confidence interval (CI), and *P* value of the association between the implementation of measures to increase patient satisfaction because of narrative comments and independent variables.

Multimedia Appendix 5 Comparison of the study sample with the registered providers on jameda and providers in the outpatient sector in Germany.

## Abbreviations

AOR: adjusted odds ratio
GP: general practitioner
PRW: physician-rating website
