# Supplementary material for: Do Health Care Providers Use Online Patient Ratings to Improve the Quality of Care? Results From an Online-Based Cross-Sectional Study
Source: J Med Internet Res. 2016 Sep 19;18(9):e254. doi: 10.2196/jmir.5889 (PMC5048057; doi:10.2196/jmir.5889)
Supplement: Multimedia Appendix 1 [file jmir_v18i9e254_app1.pdf]

Supplemental File 1: The survey

Introduction

Dear survey participant,

In the following survey, we would like to ask you about your knowledge of physician ratings websites and find out, whether and how you react on ratings given for your practice.

The questionnaire is divided into **three parts**. In the *first section* we would like to ask for socio-demographic information. In the *second section* we would like to know about your knowledge and usage of physician rating websites. The *third section* contains specific questions about the rating systems of physician rating websites and evaluates whether and how you react on online ratings.

The survey will last approximately five minutes. The gained results will be assessed by the statutory regulations on data protection and will be treated absolutely confidential and anonymous. The evaluations will be performed in a statistical and aggregated form.

Thank you in advance for your participation,
we highly appreciate your contribution.

Lottery

Among all participants we draw **four amazon vouchers with a value of 150 euro each**! Therefore you can enter your Email at the end of the survey

General_socio-demographic_information

Which jameda product are you using?
- Basic Product
- Silver
- Gold
- Platinum

At the beginning, we would like to ask you for some socio-demographic information.

Please indicate your gender.

- Male
- Female
- I wish not to answer this question.

Please indicate your year of birth. [e.g., 1984]

- Year of birth
- I wish not to answer this question.

Please indicate your marital status.

- Married
- Widowed
- Single
- Divorced
- I wish not to answer this question.

Please indicate the state of your practice.

- Baden-Württemberg
- Bavaria
- Berlin
- Brandenburg
- Bremen
- Hamburg
- Hessen
- Mecklenburg-Vorpommern
- Lower Saxony
- North Rhine-Westphalia
- Rhineland-Palatinate
- Saarland
- Saxony
- Saxony-Anhalt
- Schleswig-Holstein
- Thuringia
- I wish not to answer this question.

Please indicate the duration of your practice affiliation (in years).

- Years
- I wish not to answer this question.

Specific_socio-demographic_information

Which of the following medical/special trainings have you completed?

[multiple choices possible]

General practitioner
- General medicine
- Internal medicine
- Paediatrics and adolescent medicine

Specialists
- Anaesthesiology
- Anatomy
- Occupational medicine
- Ophthalmology
- Biochemistry
- Surgery
- Gynecology and obstetrics
- Otorhinolaryngology
- Dermatology and sexually transmitted diseases
- Human genetics
- Hygiene and environmental medicine
- Internal medicine
- Paediatrics and adolescent Medicine
- Children and adolescent psychiatry and psychotherapy
- Laboratory medicine
- Microbiology, virology and infection epidemiology
- Oral and maxillofacial surgery
- Neurosurgery
- Neurology
- Nuclear medicine
- Public health system
- Pathology
- Pharmacology
- Physical und rehabilitative medicine
- Physiology
- Psychiatry und Psychotherapy
- Psychosomatic medicine and Psychotherapy
- Radiology
- Forensic medicine
- Radiotherapy
- Transfusion medicine
- Urology
- Dentistry

Other trainings

- Midwives
- Alternative practitioner
- Speech therapy
- Others

Physician rating websites introduction

We would like to thank you for the first information. The second part includes questions about your knowledge and usage of physician rating websites.

How frequently do you use the internet?

- Several times a day
- Once per day
- Several times a week
- Once per week
- Less frequently

Have you ever heard of any of the following physician rating websites?

[multiple choices possible]

- AOK-Arztnavigator
- jameda
- Imedo
- Arzt-Auskunft
- Weisse Liste
- Sanego
- TK-Ärzteführer
- DocInsider
- Arzt Atlas
- Die Arztempfehlung
- KennstDuEinen
- Die-Endverbraucher
- Med
- yelp
- pointoo
- Others
- I don´t know any of these websites.

How did you become aware of physician rating websites?

[multiple choices possible]

- Contact with physician rating website provider
- Internet
- Contact with patients
- Recommendations by peers
- Advertisement
- Newspapers and/or magazines
- Recommendations by friends or relatives
- Others
- I don´t know any physician rating website.

How often do you use physician rating websites?

- Several times a day
- Once per day
- Several times a week
- Once per week
- Once per month
- Less frequently
- I do not use any physician rating website.

For what purpose(s) do you use physician rating websites?

[multiple choices possible]

- Reading own ratings
- Commenting on own ratings
- Receiving hints for improvement activites regarding patient satisfaction
- Reading ratings of other physicians because of interest
- Readings ratings of other physicians for patient referral
- Marketing activies
- I use physician rating websites for other purposes:
- I do not use physician rating websites.

How often do you evaluate your ratings on physician rating websites?

- Once per day
- Several times a week
- Once per week
- Several times a month
- Once per month
- Less frequently
- I have never evaluated my ratings.

Who is responsible for evaluating the online ratings for your practice?

[multiple choices possible]

- I evaluate the online ratings myself
- Medical assistant(s)
- Practice manager
- Others
- I have never evaluated my ratings.

Questions scaled rating systems

The following part contains questions about the rating systems of physician rating websites. There are mainly two different possibilities for patients: Scaled rating systems (e.g. stars, grades) and narrative comments.

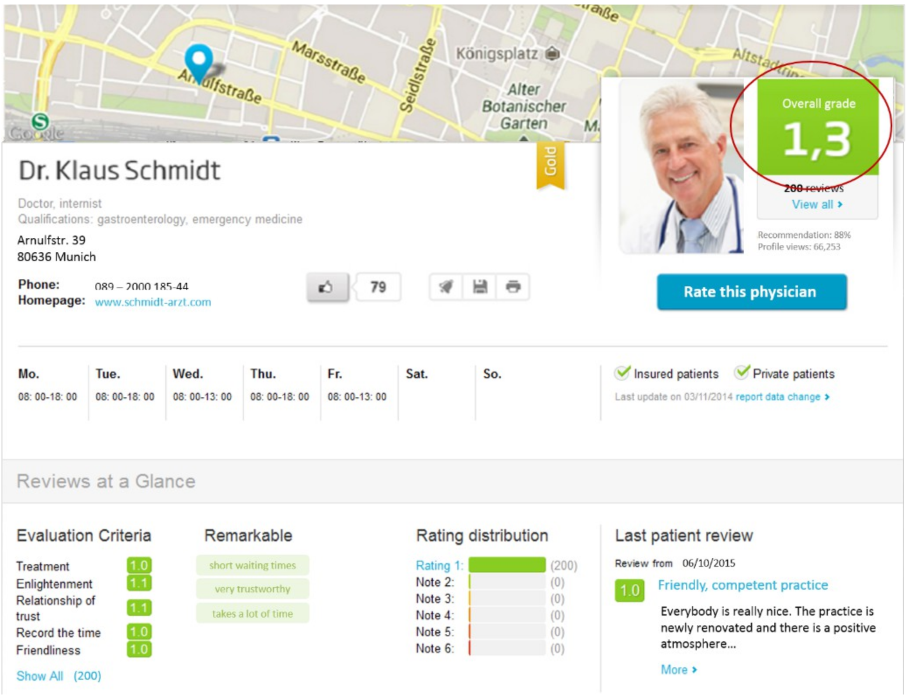

At the beginning we would like to know, whether and how you react on patients' ratings on scaled rating systems. In the following image scaled rating systems are presented with a red frame.

The patient satisfaction is rated by different evaluation criteria. Which criteria led to measures for improving patient satisfaction?

[multiple choices possible]

- Satisfaction with treatment
- Health education
- Relationship of trust
- Time spent with the patient
- Friendliness
- Waiting time to get an appointment
- Waiting time within the practice
- Office-hours
- Entertainment (e.g. magazines)
- Child friendliness
- Practice equipment
- Cleanliness
- Recommendation of other providers
- Others
- I have not implemented any measures to improve patient satisfaction because of scaled survey results.

Which measures have you implemented because of ratings on scaled rating systems (e.g. grades, stars) to increase patient satisfaction?

[multiple choices possible]

- Improve appointment scheduling process
- Change office workflow
- Expand office hours
- Reassigning staff responsibilities
- Further educational training myself
- Training of the staff
- Recruitment of additional staff
- Dismissing staff
- Patient reminders (e.g. email-reminders for preventive medical appointments)
- Introduction of guidelines
- Planing of follow-up tests
- Higher usage of guidelines
- Investments in new technologies/equipment (IT equipment)
- Improvement of the communication with other providers (e.g. physicians, hospitals)
- Hygiene improvement measures
- Improvement of the communication with patients
- Improvement of the waiting room equipment (e.g. magazines)
- Others
- I have not implemented any measures to improve patient satisfaction because of scaled survey results.

Do the ratings on scaled rating systems for your practice reflect your own expectations?

- I think that my ratings on scaled rating systems are better than I would have expected.
- I think that my ratings on scaled rating systems are similiar to what I would have expected.
- I think that my ratings on scaled rating systems are worse than I would have expected.
- I do not know my ratings.

Please note: The following question only refers to grades as one example for scaled rating systems.

At which grade do you think it is necessary to implement measures to increase patient satisfaction?

- 1
- 2
- 3
- 4
- 5
- 6
- I don't think it is necessary to implement measures to increase patient satisfaction at any grade.

Jameda questions narrative comments

In the next part we would like to know how you perceive the relevance of narrative comments. In the following image narrative comments appear with a red frame.

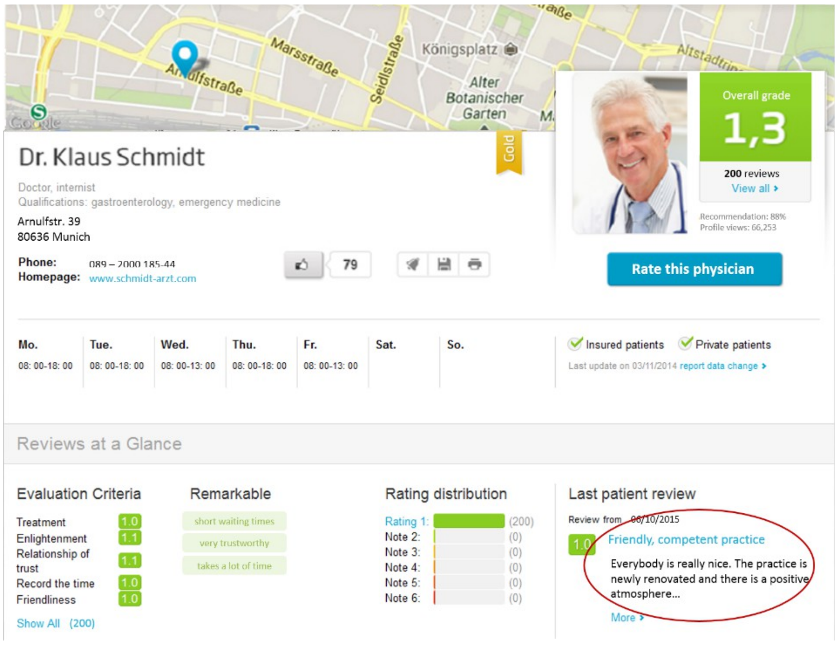

Patients address different issues in narrative comments. Which issues in narrative comments led to measures for increasing patient satisfaction?

[multiple choices possible]

- Professional competence of the physician
- Friendliness and caring attitude of the physician
- Time the physician spent for the patient
- Information and advice of the physician
- Recommendation of the physician
- Trust towards physician
- Communication between physician and patient
- Satisfaction with treatment
- Being taken seriously by the physician
- Friendliness of the office staff
- Waiting time within the practice
- Waiting time to get an appointment
- Practice equipment
- Hygiene
- Other
- I have not implemented any measures to improve patient satisfaction because of narrative comments.

Which measures have you implemented because of narrative comments to increase patient satisfaction?

[multiple choices possible]

- Improve appointment scheduling process
- Change office workflow
- Expand office hours
- Reassigning staff responsibilities
- Further educational training myself
- Training of the staff
- Recruitment of additional staff
- Dismissing staff
- Patient reminders (e.g. email-reminders for preventive medical appointments)
- Introduction of guidelines
- Planing of follow-up tests
- Higher usage of guidelines
- Investment in new technologies/practice equipment (IT equipment)
- Improvement of the communication with patients
- Improvement of the communication with other providers (e.g. physicians, hospitals)
- Hygiene improvement measures
- Improvements of the waiting room equipment (e.g. magazines)
- Others
- I have not implemented any measures to improve patient satisfaction because of narrative comments.

Do the narrative comments for your practice reflect your own expectations?

- I think that my narrative comments are better than I would have expected.
- I think that my narrative comments are similar to what I would have expectations.
- I think that my narrative comments are worse than I would have expected.
- I do not know my ratings.

Scaled rating systems narrative comments

Which ratings do you read more frequently?

- Scaled ratings (e.g. stars, grades)
- Narrative comments
- I read both equally.
- I do not read scaled ratings and narrative comments.

How trustworthy do you perceive the individual rating systems of physician rating websites?

	Not trustworthy at all				Very trustworthy
Scaled rating systems (e.g., grades, stars)	○	○	○	○	○
Narrative comments	○	○	○	○	○

Which ratings do you use more frequently to implement quality measures for increasing patient satisfaction?

- Scaled ratings (e.g. stars, grades)
- Narrative comments
- I use both equally.
- I do not use scaled ratings and narrative comments.

Websites quality measures

Which physician rating websites have led to improvement measures to increase patient satisfaction in your practice?

[multiple choices possible]

- AOK-Arztnavigator
- jameda
- Imedo
- Arzt-Auskunft
- Weisse Liste
- Sanego
- TK-Ärzteführer
- DocInsider
- Arzt Atlas
- Die Arztempfehlung
- KennstDuEinen
- Die-Endverbraucher
- Med
- yelp
- pointoo
- Others
- I haven't implemented any measures to increase patient satisfaction because of my ratings on physician rating websites.
