# Supplementary material for: Do Health Care Providers Use Online Patient Ratings to Improve the Quality of Care? Results From an Online-Based Cross-Sectional Study
Source: J Med Internet Res. 2016 Sep 19;18(9):e254. doi: 10.2196/jmir.5889 (PMC5048057; doi:10.2196/jmir.5889)
Supplement: Multimedia Appendix 2 [file jmir_v18i9e254_app2.pdf]

Medical discipline	Any measure implemented, n (%)	Mean measures, mean (SD)	Measure, n (%)																
			M1	M2	M3	M4	M5	M6	M7	M8	M9	M10	M11	M12	M13	M14	M15	M16	M17
Ophthalmology	40 (67.80%)	3.48 (2.61)	20 (33.90%)	23 (38.98%)	17 (28.81%)	13 (22.03%)	11 (18.64%)	13 (22.03%)	6 (10.17%)	6 (10.17%)	2 (3.39%)	3 (5.08%)	6 (10.17%)	2 (3.39%)	6 (10.17%)	3 (5.08%)	5 (8.47%)	0 (0.00%)	2 (3.39%)
Gynecology/obstetrics	123 (65.43%)	3.29 (2.28)	58 (30.85%)	59 (31.38%)	57 (30.32%)	26 (13.83%)	29 (15.43%)	26 (13.83%)	18 (9.57%)	22 (11.70%)	30 (15.96%)	10 (5.32%)	16 (8.51%)	8 (4.26%)	11 (5.85%)	9 (4.79%)	6 (3.19%)	4 (2.13%)	5 (2.66%)
Physical und rehabilitative medicine	15 (65.22%)	2.43 (1.83)	7 (30.43%)	3 (13.04%)	6 (26.09%)	2 (8.70%)	1 (4.35%)	2 (8.70%)	1 (4.35%)	0 (0.00%)	1 (4.35%)	4 (17.39%)	1 (4.35%)	2 (8.70%)	0 (0.00%)	0 (0.00%)	2 (8.70%)	0 (0.00%)	0 (0.00%)
Otorhinolaryngology (ENT)	59 (62.11%)	3.44 (2.42)	38 (40.00%)	25 (26.32%)	28 (29.47%)	10 (10.53%)	11 (11.58%)	13 (13.68%)	16 (16.84%)	6 (6.32%)	7 (7.37%)	10 (10.53%)	7 (7.37%)	7 (7.37%)	8 (8.42%)	5 (5.26%)	4 (4.21%)	6 (6.32%)	1 (1.05%)
Neurosurgery	17 (60.71%)	3.07 (1.62)	12 (42.86%)	10 (35.71%)	6 (21.43%)	1 (3.57%)	4 (14.29%)	2 (7.14%)	2 (7.14%)	3 (10.71%)	0 (0.00%)	1 (3.57%)	2 (7.14%)	1 (3.57%)	1 (3.57%)	1 (3.57%)	1 (3.57%)	1 (3.57%)	0 (0.00%)
Surgery/orthopedists	140 (60.61%)	3.02 (2.27)	75 (32.47%)	70 (30.30%)	70 (30.30%)	30 (12.99%)	29 (12.55%)	29 (12.55%)	20 (8.66%)	19 (8.32%)	11 (4.76%)	12 (5.19%)	10 (4.33%)	12 (5.19%)	7 (3.03%)	5 (2.16%)	9 (3.90%)	4 (1.73%)	7 (3.03%)
Oral and maxillofacial surgery	21 (60.00%)	3.45 (1.21)	10 (28.57%)	13 (37.14%)	9 (25.71%)	6 (17.14%)	3 (8.57%)	2 (5.71%)	6 (17.14%)	3 (8.57%)	2 (5.71%)	2 (5.71%)	1 (2.86%)	3 (8.57%)	0 (0.00%)	1 (2.86%)	1 (2.86%)	0 (0.00%)	1 (2.86%)
Urology	38 (58.46%)	4.29 (3.05)	25 (38.46 %)	23 (35.38%)	22 (33.85%)	8 (12.31%)	10 (15.38%)	15 (23.08%)	6 (9.23%)	6 (9.23%)	9 (13.85%)	5 (7.69%)	6 (9.23%)	6 (9.23%)	5 (7.69%)	3 (4.62%)	3 (4.62%)	4 (6.15%)	6 (9.23%)
Dentistry	350 (57.47%)	3.12 (2.45)	197 (32.35%)	127 (20.85%)	113 (18.56%)	84 (13.79%)	77 (12.64%)	61 (10.02%)	60 (9.85%)	54 (8.87%)	68 (11.17%)	39 (6.40%)	45 (7.39%)	23 (3.78%)	37 (6.08%)	27 (4.43%)	26 (4.27%)	16 (2.63%)	18 (2.96%)
Dermatology and sex. tr. diseases	41 (55.41%)	2.79 (1.91)	24 (32.43%)	21 (28.38%)	14 (18.92%)	4 (5.41%)	8 (10.81%)	8 (10.81%)	3 (4.05%)	2 (2.70%)	4 (5.41%)	5 (6.76%)	5 (6.76%)	1 (1.35%)	2 (2.70%)	5 (6.76%)	0 (0.00%)	2 (2.70%)	1 (1.35%)
Internal medicine (specialist)	45 (53.57%)	2.93 (2.10)	24 (28.57%)	26 (30.95%)	20 (23.81%)	7 (8.33%)	6 (7.14%)	9 (10.71%)	9 (10.71%)	4 (4.76%)	5 (5.95%)	4 (4.76%)	5 (5.95%)	7 (8.33%)	1 (1.19%)	1 (1.19%)	0 (0.00%)	1 (1.19%)	2 (2.38%)
General medicine	158 (52.32%)	2.94 (2.08)	81 (26.82%)	66 (21.85%)	67 (22.19%)	34 (11.26%)	31 (10.26%)	31 (10.26%)	28 (9.27%)	21 (6.95%)	20 (6.62%)	12 (3.97%)	19 (6.29%)	13 (4.30%)	2 (0.66%)	10 (3.31%)	10 (3.31%)	13 (4.30%)	8 (2.65%)
Psychosomatic medicine and Psychotherapy	18 (50.00%)	3.22 (2.44)	9 (25.00%)	7 (19.44%)	3 (8.33%)	6 (16.67%)	2 (5.56%)	3 (8.33%)	4 (11.11%)	3 (8.33%)	4 (11.11%)	3 (8.33%)	1 (2.78%)	4 (11.11%)	1 (2.78%)	1 (2.78%)	1 (2.78%)	3 (8.33%)	1 (2.78%)
Internal medicine (GP)	40 (45.45%)	3.10 (2.15)	21 (23.86%)	22 (25.00%)	22 (25.00%)	5 (5.68%)	10 (11.36%)	8 (9.09%)	4 (4.55%)	6 (6.82%)	4 (4.55%)	3 (3.41%)	5 (5.68%)	2 (2.27%)	1 (1.14%)	2 (2.27%)	1 (1.14%)	4 (4.55%)	1 (1.14%)
Alternative practitioner	120 (42.25%)	2.70 (2.14)	49 (17.25%)	38 (13.38%)	28 (9.86%)	19 (6.69%)	10 (3.52%)	7 (2.46%)	17 (5.99%)	30 (10.58%)	18 (6.34%)	37 (13.03%)	6 (2.11%)	11 (3.87%)	11 (3.87%)	4 (1.41%)	8 (2.82%)	5 (1.76%)	8 (2.82%)
Paediatrics and adolescent medicine	16 (40.00%)	1.81 (1.05)	10 (25.00%)	6 (15.00%)	3 (7.50%)	4 (10.00%)	1 (2.50%)	1 (2.50%)	0 (0.00%)	0 (0.00%)	0 (0.00%)	1 (2.50%)	0 (0.0%0)	0 (0.00%)	0 (0.00%)	1 (2.50%)	0 (0.00%)	0 (0.00%)	0 (0.00%)
Psychiatry und psychotherapy	29 (37.66%)	2.61 (1.64)	16 (20.78%)	13 (16.88%)	10 (12.99%)	6 (7.79%)	5 (6.49%)	0 (0.00%)	3 (3.90%)	6 (7.79%)	2 (2.60%)	6 (7.79%)	2 (2.60%)	1 (1.30%)	1 (1.30%)	0 (0.00%)	0 (0.00%)	2 (2.60%)	0 (0.00%)
Total	*1290 (54.66%)*	*3.06 (2.29)*	*679 (28.77%)*	*557 (23.60%)*	*501 (21.23%)*	*266 (11.27%)*	*245 (10.38%)*	*231 (9.79%)*	*200 (8.47%)*	*189 (8.01%)*	*184 (7.80%)*	*157 (6.65%)*	*139 (5.89%)*	*108 (4.58%)*	*100 (4.24%)*	*78 (3.31%)*	*77 (3.26%)*	*65 (2.75%)*	*64 (2.71%)*
M1=Improvement of the communication with patients,; M2=improve appointment scheduling process,; M3=change office workflow,; M4=improvement of the waiting room equipment,; M5=training of the staff,; M6=reassigning staff responsibilities,; M7=investments in new technologies/equipment,; M8=expand office hours,; M9=introduction of patient reminders,; M10=further educational training myself,; M11=recruitment of additional staff,; M12=improvement of the communication w./with other providers,; M13=introduction of guidelines,; M14=dismissing staff,; M15=higher usage of guidelines,; M16=planning of follow-up tests,; M17=hygiene improvement measures																			

Supplemental file 5: Overview of all measures that were implemented to improve patient care because of online ratings according to the medical specialty (medical disciplines with N>20) (N=2360)
