# Supplementary material for: Do Health Care Providers Use Online Patient Ratings to Improve the Quality of Care? Results From an Online-Based Cross-Sectional Study
Source: J Med Internet Res. 2016 Sep 19;18(9):e254. doi: 10.2196/jmir.5889 (PMC5048057; doi:10.2196/jmir.5889)
Supplement: Multimedia Appendix 3 [file jmir_v18i9e254_app3.pdf]

| Characteristics | Model 1[§] OR | Model 1[§] 95% CI | | Model 1[§] p | Model 2[$] OR | Model 2[$] 95% CI | | Model 2[$] p | Model 3[&] OR | Model 3[&] 95% CI | | Model 3[&] p |
|---|---|---|---|---|---|---|---|---|---|---|---|---|
| Age | | | | .473 | | | | .615 | | | | .674 |
| to 35 years[#] | | | | | | | | | | | | |
| 36 to 45 years | 0.82 | 0.53 | 1.27 | .367 | 0.82 | 0.53 | 1.28 | .387 | 0.91 | 0.58 | 1.44 | .689 |
| 46 to 55 years | 0.84 | 0.55 | 1.30 | .432 | 0.88 | 0.57 | 1.35 | .548 | 0.99 | 0.63 | 1.56 | .960 |
| 56 to 65 years | 0.79 | 0.50 | 1.24 | .304 | 0.83 | 0.52 | 1.31 | .420 | 0.93 | 0.58 | 1.51 | .770 |
| 66 years and older | 0.54 | 0.28 | 1.06 | .073 | 0.60 | 0.30 | 1.17 | .133 | 0.66 | 0.33 | 1.32 | .237 |
| Gender | | | | | | | | | | | | |
| Male[#] | | | | | | | | | | | | |
| Female | 1.22 | 0.99 | 1.50 | .052 | 1.18 | 0.96 | 1.45 | .114 | 1.11 | 0.90 | 1.38 | .337 |
| Marital status | | | | .124 | | | | .144 | | | | .205 |
| Married[#] | | | | | | | | | | | | |
| Widowed | 2.38 | 0.85 | 6.70 | .101 | 2.44 | 0.86 | 6.87 | .092 | 1.66 | 0.58 | 4.77 | .349 |
| Single | 1.25 | 0.94 | 1.64 | .122 | 1.24 | 0.94 | 1.64 | .134 | 1.31 | 0.97 | 1.76 | .076 |
| Divorced | 1.22 | 0.86 | 1.72 | .262 | 1.17 | 0.83 | 1.66 | .374 | 1.22 | 0.84 | 1.76 | .301 |
| Internet use | | | | .549 | | | | .727 | | | | .396 |
| Several times a day[#] | | | | | | | | | | | | |
| Once a day | 1.00 | 0.68 | 1.47 | .997 | 1.06 | 0.72 | 1.56 | .766 | 1.32 | 0.87 | 1.98 | .191 |
| Less than once a day | 0.76 | 0.46 | 1.25 | .274 | 0.83 | 0.50 | 1.38 | .471 | 1.14 | 0.66 | 1.98 | .644 |
| Medical specialty | | | | <.001 | | | | .003 | | | | <.001 |
| General practitioner[#] | | | | | | | | | | | | |
| Specialist | 1.29 | 1.01 | 1.63 | .038 | 1.14 | 0.90 | 1.46 | .283 | 1.15 | 0.89 | 1.48 | .292 |
| Others | 0.78 | 0.55 | 1.09 | .143 | 0.69 | 0.49 | 0.98 | .035 | 0.60 | 0.42 | 0.87 | .007 |
| Jameda product | | | | | | | | | | | | |
| Basic product# | | | | | | | | | | | | |
| Any service product (e.g., gold, silver, platin) | | | | | 1.56 | 1.28 | 1.91 | <.001 | 1.10 | 0.89 | 1.37 | .381 |
| Use of physician rating websites (frequency) | | | | | | | | | | | | <.001 |
| At least once per day[#] | | | | | | | | | | | | |
| Several times a week | | | | | | | | | 1.33 | 0.84 | 2.11 | .226 |
| Once per week | | | | | | | | | 0.97 | 0.64 | 1.48 | .888 |
| Once per month | | | | | | | | | 0.55 | 0.36 | 0.84 | .006 |
| Less frequently | | | | | | | | | 0.39 | 0.25 | 0.61 | <.001 |
| Never | | | | | | | | | 0.19 | 0.10 | 0.36 | <.001 |
| Appraisal of the trustworthiness of scaled-rating results | | | | | | | | | | | | .003 |
| Not at all trustworthy[#] | | | | | | | | | | | | |
| Not trustworthy | | | | | | | | | 2.07 | 1.20 | 3.55 | .009 |
| More or less trustworthy | | | | | | | | | 2.50 | 1.46 | 4.29 | <.001 |
| Somewhat trustworthy | | | | | | | | | 2.47 | 1.42 | 4.31 | <.001 |
| Very trustworthy | | | | | | | | | 1.49 | 0.76 | 2.95 | .250 |
| Appraisal of the trustworthiness of narrative comments | | | | | | | | | | | | .245 |
| Not at all trustworthy[#] | | | | | | | | | | | | |
| Not trustworthy | | | | | | | | | 1.85 | 1.04 | 3.27 | .035 |
| More or less trustworthy | | | | | | | | | 1.59 | 0.90 | 2.80 | .111 |
| Somewhat trustworthy | | | | | | | | | 1.5 | 0.8 | 2.7 | .136 |

| | | | | | | |
|---|---|---|---|---|---|---|
| Very trustworthy | | | 4<br>1.8<br>2 | 7<br>0.9<br>8 | 3<br>3.4<br>1 | .060 |
| Statistical model details | χ2(12)=30,595, p=.002<br>$R^2$(Nagelkerke)=.021 | χ2(13)=50,376, p<.001<br>$R^2$(Nagelkerke)=.034 | χ2(26)=244,788, p<.001<br>$R^2$(Nagelkerke)=.156 | | | |

[#] 1.00 (Reference category)

Note: *p<0.05, ** p<0.001

[§] Model 1: Adjusted for demographics (age, gender, marital status, internet use, medical specialty)

[$] Model 2: Adjusted for demographics, jameda product

[&] Model 3: Adjusted for demographics, jameda product, use of PRWs, appraisal of the trustworthiness of scaled-rating results/narrative comments

**Supplement file 2:** Multivariate regression analyses; adjusted odds ratio (OR), 95% confidence interval (CI), and p-value of the association between the implementation of measures to increase patient satisfaction because of scaled survey online ratings and independent variables
