# Supplementary material for: Do Health Care Providers Use Online Patient Ratings to Improve the Quality of Care? Results From an Online-Based Cross-Sectional Study
Source: J Med Internet Res. 2016 Sep 19;18(9):e254. doi: 10.2196/jmir.5889 (PMC5048057; doi:10.2196/jmir.5889)
Supplement: Multimedia Appendix 4 [file jmir_v18i9e254_app4.pdf]

Characteristics	Model 1[§] OR	95% CI		p	Model 2[$] OR	95% CI		p	Model 3[&] OR	95% CI		p
Age				.509				.535				.478
to 35 years[#]												
36 to 45 years	0.86	0.56	1.34	.508	0.87	0.56	1.35	.525	0.97	0.61	1.53	.885
46 to 55 years	0.96	0.62	1.48	.860	1.00	0.65	1.54	.991	1.14	0.72	1.79	.578
56 to 65 years	0.90	0.57	1.42	.638	0.94	0.59	1.49	.784	1.08	0.67	1.74	.764
66 years and older	0.61	0.31	1.21	.155	0.66	0.33	1.33	.245	0.74	0.36	1.50	.402
Gender												
Male[#]												
Female	1.14	0.93	1.40	.220	1.10	0.89	1.36	.366	1.04	0.84	1.30	.711
Marital status				.278				.270				.477
Married[#]												
Widowed	2.32	0.84	6.41	.104	2.38	0.86	6.56	.095	1.70	0.60	4.76	.317
Single	1.18	0.89	1.56	.257	1.17	0.88	1.55	.281	1.20	0.90	1.62	.218
Divorced	1.04	0.73	1.48	.848	1.00	0.70	1.42	.983	1.01	0.69	1.46	.974
Internet use				.726				.687				.207
Several times a day[#]												
Once a day	1.12	0.76	1.65	.575	1.18	0.80	1.75	.408	1.40	0.92	2.11	.113
Less than once a day	0.87	0.52	1.45	.590	0.95	0.57	1.59	.836	1.29	0.74	2.25	.368
Medical specialty				<.001				<.001				<.001
General practitioner[#]												
Specialist	1.21	0.95	1.54	.117	1.09	0.85	1.39	.508	1.09	0.84	1.40	.533
Others	0.55	0.38	0.79	<.001	0.49	0.34	0.71	<.001	0.43	0.29	0.63	<.001
Jameda product												
Basic product[#]												
Any service product (e.g., gold, silver, platin)					1.50	1.23	1.83	<.001	1.10	0.88	1.37	.390
Use of physician rating websites (frequency)												<.001
At least once per day[#]												
Several times a week									1.21	0.78	1.89	.397
Once per week									0.96	0.63	1.44	.826
Once per month									0.61	0.40	0.92	.019
Less frequently									0.46	0.30	0.70	<.001
Never									0.20	0.10	0.40	<.001
Appraisal of the trustworthiness of scaled-rating results												.135
Not at all trustworthy[#]												
Not trustworthy									1.47	0.84	2.55	.177
More or less trustworthy									1.75	1.01	3.02	.047
Somewhat trustworthy									1.62	0.92	2.85	.093
Very trustworthy									1.12	0.56	2.23	.757
Appraisal of the trustworthiness of narrative comments												.042
Not at all trustworthy[#]												
Not trustworthy									2.26	1.24	4.10	.008
More or less trustworthy									2.19	1.21	3.96	.010
Somewhat trustworthy									2.5	1.4	4.6	.002

Very trustworthy			5<br>2.5<br>1	0<br>1.3<br>1	2<br>4.7<br>9	.005
Statistical model details	χ2(12)=38,568, p<.001<br>R^2(Nagelkerke)=.026	χ2(13)=54,534, p<.001<br>R^2(Nagelkerke)=.037	χ2(26)=209,320, p<.001<br>R^2(Nagelkerke)=.136			

[#] 1.00 (Reference category)

Note: *p<0.05, ** p<0.001

[§] Model 1: Adjusted for demographics (age, gender, marital status, internet use, medical specialty)

[$] Model 2: Adjusted for demographics, jameda product

[&] Model 3: Adjusted for demographics, jameda product, use of PRWs, appraisal of the trustworthiness of scaled-rating results/narrative comments

Supplemental file 3: Multivariate regression analyses; adjusted odds ratio (OR), 95% confidence interval (CI), and p-value of the association between the implementation of measures to increase patient satisfaction because of narrative comments and independent variables
