# Supplementary material for: Do Health Care Providers Use Online Patient Ratings to Improve the Quality of Care? Results From an Online-Based Cross-Sectional Study
Source: J Med Internet Res. 2016 Sep 19;18(9):e254. doi: 10.2196/jmir.5889 (PMC5048057; doi:10.2196/jmir.5889)
Supplement: Multimedia Appendix 5 [file jmir_v18i9e254_app5.pdf]

Characteristics	Study sample (N=2,360)	Registered providers on jameda	Providers in the German outpatient sector (2014)
Medical specialty[1]			
General practitioners	17.5%	20.1%	17.8%
Specialists	69.4%	59.5%	53.5%
Others	13.1%	20.4%	28.7%
Age[2]			
Mean	49.6	56.9	53.4
Gender[2]			
Male	66.7%	62.7%	57.6%
Female	33.3%	37.3%	42.4%
State[2]			
Baden-Wuerttemberg	12.9%	12.6%	13.3%
Bavaria	19.9%	20.4%	17.4%
Berlin	6.3%	6.7%	5.2%
Brandenburg	2.1%	1.9%	2.5%
Bremen	0.7%	0.8%	1.1%
Hamburg	3.1%	3.4%	3.2%
Hesse	9.4%	8.4%	7.1%
Mecklenburg-Western Pomerania	0.8%	1.1%	1.8%
Lower Saxony	6.0%	6.9%	8.9%
North Rhine-Westphalia	23.8%	23.3%	21.1%
Rhineland-Palatinate	4.3%	4.0%	4.6%
Saarland	1.1%	0.9%	1.3%
Saxony	3.9%	3.6%	4.5%
Saxony-Anhalt	1.3%	1.5%	2.3%
Schleswig-Holstein	2.7%	3.1%	3.5%
Thuringia	1.3%	1.5%	2.3%

[1] Related to all providers in the outpatient sector.

[2] The column “Providers in the German outpatient sector” is related to all physicians in the outpatient sector (without dentists)

Supplemental file 4: Comparison of the study sample with the registered providers on jameda and providers in the outpatient sector in Germany
